# Banxia xiexin decoction and its bioactive metabolites: multi-targeted mechanisms for suppressing gastric cancer progression, reversing chemoresistance, and remodeling the tumor microenvironment

**DOI:** 10.3389/fphar.2026.1810848

**Published:** 2026-06-17

**Authors:** Donghan Xu, Ziqing Lin, Lu Deng, Xiaonan Lu, Ziyi Guo, Peiyu Yan, Yehao Luo

**Affiliations:** 1 Department of Endocrinology, The Second Affiliated Hospital of Guangzhou University of Chinese Medicine, Guangzhou, China; 2 Department of Endocrinology, Guangdong Provincial Hospital of Chinese Medicine, Guangzhou, China; 3 Faculty of Chinese Medicine, Macau University of Science and Technology, Macao, Macao SAR, China; 4 Jianyang People’s Hospital, Chengdu, China; 5 Wuming Hospital of Guangxi Medical University, Nanning, China; 6 National Engineering Laboratory for Internet Medical Systems and Applications, The First Affiliated Hospital of Zhengzhou University, Zhengzhou, China; 7 Department of Rehabilitation Medicine, The First Affiliated Hospital of Zhengzhou University, Zhengzhou, China

**Keywords:** active components, Banxia xiexin decoction, gastric cancer, mechanisms of action, multi-target therapy

## Abstract

Gastric cancer (GC) remains a leading cause of cancer-related mortality worldwide, primarily due to late diagnosis and limited benefit from surgery alone. Although chemotherapy, targeted agents, and immunotherapy have improved outcomes for selected patients, their clinical benefits are often limited by significant toxicity, acquired resistance, and the pronounced molecular heterogeneity of GC. Multi-target therapeutic approaches are therefore urgently needed. Banxia Xiexin Decoction (BXD), a classic Traditional Chinese Medicine formula widely used for gastrointestinal disorders, has emerged as a promising adjuvant candidate for GC treatment. However, the bioactive metabolites and molecular mechanisms of BXD have not been fully clarified. This review comprehensively summarizes current evidence on the anti-GC actions of BXD and its key bioactive metabolites. Mechanistically, BXD inhibits GC cell proliferation and induces apoptosis by regulating cell-cycle checkpoints and inhibiting oncogenic pathways, particularly Wnt/β-catenin and the PI3K/AKT/mTOR axis. These coordinated effects facilitate apoptosis, autophagy modulation, and oxidative stress–related cytotoxicity, and are further linked to reduced epithelial–mesenchymal transition (EMT), invasion, migration, and angiogenesis. The major bioactive metabolites of BXD, such as berberine, baicalin, wogonoside, and glycyrrhizin further reverse chemoresistance by downregulating drug-efflux and survival signaling, thereby enhancing sensitivity to standard agents such as cisplatin, 5-fluorouracil, oxaliplatin, and paclitaxel. BXD also shows potential in suppressing peritoneal metastasis by disrupting pre-metastatic niche formation and in improving anti-tumor immunity through downregulation of PD-L1 via the IL-6/JAK/STAT3 pathway, reduction of immunosuppression, and promotion of immunogenic cell death (ICD). Furthermore, BXD-associated regulation of metabolic reprogramming (e.g., GSK3β and HNF4α) may undermine GC cellular adaptability under therapeutic stress. These findings highlight BXD as a promising multi-component, multi-pathway adjuvant candidate for GC, exerting cooordinated effects on tumor cell survival, metastasis, drug resistance, metabolism, and immune regulation. Nevertheless, limitations of current studies include insufficient investigation of tumor microenvironmental (TME) components (particularly macrophages, exosomes, and mesenchymal stem cells) and a lack of standardized pharmacokinetic/pharmacodynamic characterization (PK/PD). Future research should integrate multi-omics, spatial transcriptomics, and rigorous preclinical and clinical trials to improve reproducibility, clarify active metabolite–target relationships, elucidate BXD-mediated remodeling of the GC TEM to enhance therapeutic responsiveness.

## 1 Introduction

Gastric cancer (GC) is a globally prevalent malignancy with high mortality rates (Thrift et al., 2023; Yu et al., 2024). Due to the lack of effective early diagnostic tools, most patients are diagnosed at an advanced stage (Li et al., 2026; Wang et al., 2021), at which point the efficacy of surgery, the only potentially curative treatment, is limited (Lee, 2026; Sundar et al., 2025). Consequently, there is an urgent need for effective adjuvant therapies (Ji et al., 2024).

While current adjuvant therapies, including chemotherapy, targeted therapy, and immune-otherapy, have achieved some progress, their clinical benefits are often hampered by significant toxicity, acquired drug resistance, and limited applicability to only a subset of patients (Chen et al., 2020; Digklia and Wagner, 2016; Wu et al., 2025; Xu et al., 2023; Zhang et al., 2026). The marked heterogeneity of GC, in particular, renders single-target therapies insufficient for sustained tumor control (Chen et al., 2020; Chen et al., 2025; Lei et al., 2022). Therefore, therapies targeting multiple targets and pathways have emerged as a major focus of current research.

Traditional Chinese Medicine (TCM) formulas, characterized by their multi-component and multi-target nature, offer a novel therapeutic paradigm for GC (Liao et al., 2023; Liu Y. et al., 2024). BXD, a classical formula for treating gastrointestinal diseases, has demonstrated considerable anti-tumor potential (Zhang et al., 2021). The formula was first recorded in the *Shanghan Lun* (Treatise on Cold Damage Diseases) during the Han Dynasty, and it is composed of seven botanical drugs including *Pinellia ternata* [Thunb.] Makino [Araceae], *Scutellaria baicalensis* Georgi [Lamiaceae], *Coptis chinensis* Franch. [Ranunculaceae], *Zingiber officinale* Roscoe [Zingiberaceae], *Panax ginseng* C.A. Mey. [Araliaceae], *Ziziphus jujuba* Mill. [Rhamnaceae], and *Glycyrrhiza glabra* L. [Fabaceae]. All botanical names and species have been verified through the Medicinal Plant Names Services (MPNS) portal (http://mpns.kew.org/mpns-portal/).

However, due to its complex composition, the underlying mechanisms of action of BXD and its active metabolites against GC remain to be fully elucidated. This review aims to systematically summarize these mechanisms, thereby providing a comprehensive reference to further clinical and experimental studies.

## 2 Methods

### 2.1 Search strategy

A comprehensive literature search was conducted on the use of BXD for GC treatment. Relevant studies published from the inception of each database up to February 2026 were retrieved from multiple electronic databases, including PubMed, Web of Science, Google Scholar, Cochrane Library, China National Knowledge Infrastructure (CNKI), and Wanfang Database. The following keywords were used for literature search: (“gastric cancer” OR “gastric carcinoma” OR “gastric neoplasm” OR “stomach carcinoma” OR “stomach neoplasm” OR “stomach cancer”) AND (“Ban xia xie xin” OR “Banxia xiexin” OR “Banxia xiexin decoction” OR “Banxia Xiexin Tang” OR “Banxia Xiexin metabolite”).

### 2.2 Inclusion and exclusion criteria

The inclusion criteria were as follows: (1) clinical studies, *in vivo*, or *in vitro* experimental studies evaluating the therapeutic effects of BXD on GC; (2) studies in which the experimental group involved BXD intervention for GC, with no restrictions on dosage, frequency, or route of administration; (3) no restrictions on the species, sex, age, or body weight of experimental animals, or on the cell lines studied; and (4) the research topic was GC.

The exclusion criteria were as follows: (1) duplicate publications, review articles, or studies unrelated to the topic; (2) studies with incomplete outcome data; and (3) studies for which the full text was unavailable.

## 3 Clinical application of BXD in the treatment of GC

BXD and chemotherapy combination has demonstrated multifaceted clinical benefits in the treatment of postoperative and advanced GC patients. This combination therapy can significantly boost patients’ immune function, improve chemotherapy completion rates, and enhance quality of life, with safety and tolerability (Feng and Zhang, 2022; Gao, 2020; Ni, 2014; Yang, 2023; Zhang, 2022). In patients with advanced GC, the regimen effectively alleviated chemotherapy-induced gastrointestinal adverse reactions and improved treatment tolerance (Dai, 2020; Li and Wang, 2019; Qiu, 2019). In addition, studies have shown that the combination therapy regulated multiple serum biomarkers, including reduction of hypoxia-inducible factor-1α, monocyte chemoattractant protein-1, and endothelin-1 levels (Feng and Zhang, 2022), as well as improvement of tumor markers (Tan, 2021). Regarding survival outcomes, the combination could prolong median progression-free survival and reduce the risk of postoperative recurrence (Liu, 2020; Tao, 2013; Zhou, 2014).For postoperative gastric stasis in GC patients, BXD effectively relieved symptoms such as abdominal distension, nausea, and vomiting, and reduced gastric tube drainage volume (Xing, 2017).

## 4 Intervention mechanisms of BXD in the onset and progression of GC

**FIGURE 1:**
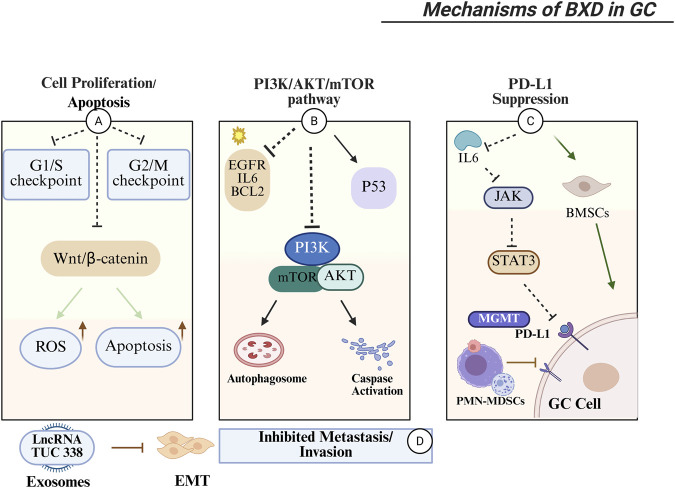
Intervention mechanisms of BXD in the onset and development of GC (Created in https://BioRender.com).

BXD inhibits GC progression through multiple mechanisms. It inhibits cell proliferation and induces apoptosis by targeting cell cycle checkpoints (G1/S and G2/M) (Tomasello et al., 2016) and inhibiting the Wnt/β-catenin pathway, thereby promoting oxidative stress-induced apoptosis (Sun et al., 2021). Additionally, BXD downregulates lncRNA TUC338, which inhibits GC cell invasion, migration, and EMT (Dai et al., 2023) (see Figure 1).

Much evidence indicates that BXD interferes with the PI3K/AKT/mTOR signaling axis, a central driver of GC progression. Multi-omics analyses have confirmed that BXD downregulates critical components of this pathway, including PI3K subunits, AKT, mTOR, and related factors such as EGFR, PIK3CA, IL6, BCL2, AKT1, HSP90, and p53 (Li et al., 2024; Zu et al., 2024; Zu et al., 2025). This modulation inhibits cell proliferation while promoting autophagy and apoptosis in GC cells.

BXD also demonstrates potential in suppressing peritoneal metastasis. Prior to metastasis, GC-derived exosomes can transfer bioactive molecules to peritoneal mesothelial cells, inducing EMT and creating a pre-metastatic niche (Li et al., 2023; Ng et al., 2025; Sun et al., 2022). By interfering with these exosome-mediated processes, BXD may effectively limit peritoneal dissemination of GC cells.

Polymorphonuclear myeloid-derived suppressor cells (PMN-MDSCs) are immature, immune-suppressive cells that accumulate in the TME of GC, promoting tumor progression and metastasis through immunosuppression and the secretion of soluble factors (Gabrilovich and Nagaraj, 2009; Hu et al., 2022).

Bone marrow mesenchymal stem cells (BMSCs) are currently being investigated as potential carriers for targeted GC therapy because of their tumor-tropic migration capacity, ease of isolation, and genetic modifiability (Nakamizo et al., 2005; Studeny et al., 2004).

Furthermore, BXD exerts anti-GC effects, in part, by downregulating PD-L1 expression. It modulates PD-L1 via the IL-6/JAK/STAT3 pathway, which can enhance chemosensitivity to cisplatin by regulating MGMT activity (Feng et al., 2021b). BXD also inhibits GC cell proliferation and induces apoptosis through multi-target suppression of PD-L1 (Feng et al., 2021a) (see Table 1).

**TABLE 1:** Summary of BXD intervention mechanisms for GC.

*In vivo* or *in vitro* experiments/Pharmaceutical manufacturing method/Dosage/intervention time	Species	Group	Target	Mechanism	References
*In vitro*/BXD serum containing medicine/25,50,100 μL/mL/-	SNU-16	Control group (SNU-16 cells were cultured without any treatment),BXD + LiCl group (SNU-16 cells were cultured with 25 μL BXD medicated serum and 25 μL LiCl)	-	Suppresses Wnt/β-catenin signaling pathway to inhibit GC cell proliferation and colony formation, promotes oxidative stress, and induces apoptosis	Sun et al. (2021)
*In vitro*/BXD water extract/0.50,1.00,1.50 mg/mL/-	AGS, GES-1	Control AGS cells (no drug added),cells to be treated with 5-FU (2 mg/mL)	E-cadherin↑ N-cadherin↓ Vimentin↓	Suppresses GC cell migration and invasion by inhibiting the expression of lncRNA TUC338, and inhibits epithelial-mesenchymal transition	Dai et al. (2023)
*In vitro* + *In vivo*/BXD serum containing medicine/9 g/kg/5w	AGS, NCI-N87, BALB/c	AGS/DDP,15% BXD,15% BXD + covlivelin,15% BXD + DDP,15% BXD + DDP + covlivelin; AGS/DDP, DDP,BXD, DDP + BXD	PD-L1↓, MGMT↓ STAT3↓ IL6↓, JAK1↓ IFNR↓	Regulates MGMT expression to affect drug sensitivity of GC cells	Feng et al. (2021b)
*In vitro* + *In vivo*/BXD serum containing medicine/9 g/kg/5w	AGS, BALB/c	BXXX-0%,BXD-5%,BXD-10%,BXD-20%; BXD-low, BXD + Mid, BXD + High	PD-L1↓, HIF-1↓ EGFR↓, TLR4↓ IFNGR-	Inhibits PD-L1 expression through multiple targets and pathways to affect GC cell proliferation and induce apoptosis	Feng et al. (2021a)
*In vitro* + *In vivo*/BXD serum containing medicine/16.25 g/kg,32.5 g/kg,65 g/kg/2w	AGS, BALB/c	Control,the capecitabine group and the low, medium, and high dose groups of BXD	PI3K/AKT	Inhibited tumor growth and slowed cancer progression by suppressing five factors in the PI3K/AKT signaling pathway	Zu et al. (2025)
*In vitro* + *In vivo*/BXD serum containing medicine, 21.42 g/kg,42.84 g/kg, 85.68 g/kg/2w	GES-1,MGC-803, BALB/c	Control,the capecitabine group and the low, medium, and high dose groups of BXD	PI3K/AKT	BXD has the effect of inhibiting tumor growth rate and delaying the development of GC. Its mechanism of action may be related to the regulation of PI3K-Akt signaling pathway	Zu et al. (2024)
*In vitro*/BXD serum containing medicine/2.46 g/mL/-	MKN45, AGS	Control, capecitabine, low-dose BXD (serum containing 5% BXD), medium-dose BXD (serum containing 10% BXD), and high-dose BXD (serum containing 20% BXD)	PI3K/Akt/mTOR BAX, BCL-2 AKT, Beclin1 LC3, P62	BXD slows GC cell growth, encourages cell death, and triggers autophagy by controlling the PI3K/Akt/mTOR signaling pathway and important proteins involved in apoptosis and autophagy	Li et al. (2024)

## 5 The role of BXD active metabolites in the treatment of GC

**FIGURE 2:**
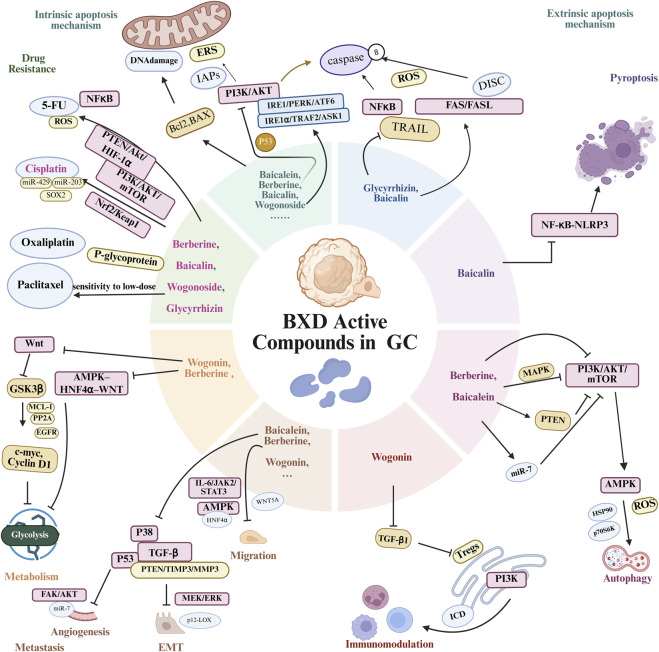
BXD active metabolites in the treatment of GC (Created in https://BioRender.com).

Ultra-performance liquid chromatography-tandem mass spectrometry (UPLC-MS/MS) was used to analyze the chemical fingerprint of BXD. Multiple major metabolites including flavonoids such as baicalin (m/z 447.1 → 271.1), baicalein (m/z 271.1 → 122.8), and wogonin (m/z 285.1 → 270.0); alkaloids such as berberine (m/z 336.0 → 336.0) and coptisine (m/z 320.0 → 292.0); and saponins such as ginsenoside Rg1 (m/z 823.5 → 643.3) and glycyrrhizic acid (m/z 845.0 → 669.0) were identified. In addition, trace amounts of bioactive metabolite such as oroxylin A were also detected. The observed variations in metabolites concentration among different decoction batches may be attributed to the formation of insoluble complexes between alkaloids and flavonoids or saponins during the decoction process (Wang et al., 2014). Furthermore, a previous study identified 95 metabolites in the aqueous extract of BXD, among which the main active metabolites (OB ≥ 30% and DL ≥ 0.18) included palmatine chloride (m/z 351.14714), berberine (m/z 335.11604), and baicalin (m/z 446.08518) (Dai et al., 2023). These are considered the primary active metabolites responsible for the anti-GC effects of BXD (see Figure 2).

The above findings provide a theoretical foundation for the multi-target mechanisms of the key active metabolites of BXD in GC, and the regulatory pathways and key targets of these validated active metabolites are elucidated in the following sections.

### 5.1 Inducing apoptosis and pyroptosis of GC cells

Apoptosis is a programmed cell death process mediated by specific proteins, particularly caspases. When activated by internal or external stimuli, caspases initiate an irreversible proteolytic cascade (Asadi et al., 2022). This process is regulated by multiple proteins and genes, including caspases, the inhibitor of apoptosis protein (IAP) family, Bax/Bcl-2 family proteins, CDKs, and p53 (Xu et al., 2024).

A key apoptotic pathway is the intrinsic (mitochondrial) pathway. Active metabolites in BXD increase mitochondrial membrane permeability, result in the release of cytochrome c into the cytoplasm (Zhao et al., 2023). Released cytochrome c then binds with Apaf-1, procaspase-9, and ATP to form the apoptosome, which activates caspase-9 and subsequently trigger downstream effector caspases such as caspase-3, -6, -7, ultimately executing apoptosis (Sun et al., 2013). This pathway is tightly regulated by the Bcl-2 protein family, which includes pro-apoptotic members (e.g., Bax, Bad, Bak) and anti-apoptotic members (e.g., Bcl-2, Bcl-xL) (Gryko et al., 2014).

Multiple natural metabolites demonstrate anti-GC effects by inducing apoptosis through distinct molecular mechanisms. Several metabolites promote apoptosis via the mitochondrial pathway. Baicalein downregulates MDM2, leading to p53 activation, which subsequently inhibits IAP family proteins (cIAP1, cIAP2, XIAP) and triggers caspase-dependent apoptosis (Gowda Saralamma et al., 2018; Mu et al., 2016). Berberine regulates AKT-related mitochondrial pathways, reducing mitochondrial membrane potential through p53 activation and subsequent caspase activation (Lin et al., 2006; Yi et al., 2015). The release of mitochondrial proteins such as Smac/DIABLO inactivates IAPs, thereby promoting caspase-9 activation (Zangemeister-Wittke and Simon, 2004). Glycyrrhetinic acid induces cell cycle arrest and apoptosis primarily by regulating the PI3K/AKT pathway (Wang et al., 2020). Glycyrrhizin promotes apoptosis and inhibits metastasis by regulating the ICMT/Ras pathway (Ma et al., 2022).

The extrinsic apoptotic pathway is activated by ligands of the TNF receptor superfamily, result in the activation of the death-inducing signaling complex (DISC) containing FADD, TRADD, and pro-caspase-8, which leads to the activation of caspase-8 and -10. (Tian et al., 2012; Zhou et al., 2020). Activated caspase-8 can directly execute apoptosis or amplify the apoptotic signal via the mitochondrial pathway (Liamarkopoulos et al., 2011). For example, glycyrrhizin synergizes with TRAIL to enhance reactive oxygen species (ROS) generation and tumor cell death (Xie et al., 2017).

Persistent endoplasmic reticulum stress (ERS) activates apoptotic pathways via IRE1, PERK, and ATF6 (Kim and Kim, 2018; Ren et al., 2020; Wang Y. et al., 2019). Wogonoside induces ERS-associated apoptosis by activating the IRE1α-TRAF2-ASK1 pathway (Gu et al., 2021). Baicalin triggers ERS and apoptosis by inhibiting the PI3K/AKT pathway (Shen et al., 2023).

Beyond apoptosis, some metabolites can induce other forms of regulated cell death. Baicalin for instance, orchestrates GC cell pyroptosis through the NF-κB-NLRP3 signaling pathway, offering an alternative therapeutic strategy (Liu J. et al., 2024) (see Table 2).

**TABLE 2:** Summary of BXD active metabolites intervention mechanisms for GC.

*In vivo* or *in vitro* experiments/Pharmaceutical manufacturing method/Dosage/Dissolving agent/intervention time	Species	Group	Target	Mechanism	References
*In vitro*/Baicalein/25,50,100μM/−/−	AGS, SNU-484	Control,25 μM; 50 μM; 100 μM	MDM2↓, p53↑, cIAP1↓, cIAP2↓, XIAP↓, bcl-xl↓, bax↑, p-caspase9↑, cleaved caspase9↑, p-caspase3↑, cleaved caspase3↑, PARP-, cleaved PARP↑	Downregulate MDM2 and activate tumor suppressor protein p53, subsequently downregulate IAP family proteins (cIAP1, cIAP2, and XIAP), significantly inhibit cell proliferation, and induce caspase-dependent cell apoptosis	Gowda Saralamma et al. (2018)
*In vitro* + *In vivo*/Baicalein/0,15,30,60 μmol/L,15,50 mg/kg/-/4w	SGC-7901	0 μmol/L,15 μmol/L 30 μmol/L 60 μmol/L,Control, low dose, high dose	Bcl-2↓, BAX↑, mitochondrial membrane potential↓, PARP↑	Induce GC cell apoptosis through the mitochondrial pathway	Mu et al. (2016)
*In vitro* + *In vivo*/Baicalein/30 μM, 15,50 mg/kg/day/-/4w	HGC-27, AGS, nude mice	blank group, DMSO group, Bai (15,30,60 µM) group, Bai (30 µM)+DMSO group, Bai (30 µM)+4-PBA group, Bai (30 µM)+siRNA-NC group Bai (30 µM)+siRNA-BTG3 group, Bai (30 µM)+siRNA-BTG3+DMSO group, and Bai (30 µM)+siRNA-BTG3+LY294002 group	Grp78↑, CHOP↑, Ca2+↑, PI3K-, p-PI3K↓, AKT-, p-AKT↓	Activate BTG3 to inhibit the PI3K/AKT pathway, trigger ER stress and induce apoptosis	Shen et al. (2023)
*In vitro*/Baicalein/50 μmol/L/−/−	BGC-823	Control group, miR-7 group, BAI group	microRNA-7-5p, p-PI3K↓, p-AKT↓, p-mTOR↓	Mediated miR-7 inhibits the formation of autophagosomes in BGC-823 cells by inhibiting PI3K/AKT/mTOR signaling pathway, and inhibits the generation of autophagic flow	Wen et al. (2024)
*In vitro*/Baicalein/0,25,50μM/−/−	AGS	0 μM,25μMor 50 μM	TGF-β↓, Smad4↓, N-cadherin↓, vimentin↓, ZEB1↓, ZEB2↓	Inhibit GC cell metastasis by suppressing TGF-β/Smad4 signaling	Chen et al. (2014)
*In vitro*/Baicalein/0, 10, 20,40 μM/−/−	MGC-803	Control group, Baicalein group	MMP2↑, MMP9↑, P38-, p-P38↓	Inhibit p38 signaling pathway to reduce cell motility and migration to inhibit GC cell invasion and metastasis	Yan et al. (2015)
*In vitro* + *In vivo*/Baicalein/0,5,15,25 μmol/L,50 mg/kg/-/3w	HGC-27, SGC-7901, MGC-803, BGC-823	0 μmol/L, 5 μmol/L, 15 μmol/L, 25 μmol/L, NC, shFAK, shFAK + MC, shFAK + BAI, Vector, FAK, FAK + MC, FAK + BAI	miR-7↑, FAK↓, PI3K-, p-PI3K↓, AKT-, p-AKT↓, vegf↓	Mediate the miR-7/FAK/AKT signaling pathway to inhibit GC cell proliferation, migration, and angiogenesis	Qiao et al. (2022)
*In vitro* + *In vivo*/Baicalein/5,10,25 μM,50 mg/kg/-/3w	MKN-74, MGC-803, GES-1	Control groups,5 μM,10 μM,25 μM Control groups and BAI groups	p12-LOX, ERK1/2, MEK1/2	Inhibit on tumor cell migration and invasion, through regulation of *p*12-LOX modulated epithelial-mesenchymal transformation	Ye et al. (2024)
*In vitro*/Baicalein/0,12.5,25,50,100,200 μM	SGC-7901, SGC-7901/DDP, MGC-803, HGC-27	0 μM,12.5 μM,25 μM,50 μM,100 μM,200 μM	LC3A/B↑, P62↓, P65↓, p-mTOR↓, p-IκBα↑, AKT↓, Nrf2↑, Keap↓, MDR1↓	Induce apoptosis and autophagy through Akt/mTOR and Nrf2/Keap1 pathways, enhance the sensitivity of GC cell SGC-7901/DDP to DDP	Li et al. (2020)
*In vitro*/Baicalein/0,20,40μM/−/−	AGS	0 μM(Normoxia), 0 μM(Hypoxia), 10 μM(Hypoxia), 20 μM(Hypoxia), 40 μM(Hypoxia)	HK2↓, LDH-A↓, PDK1↓, PTEN↑, HIF-1α↓, AKT-, p-AKT↓	Modulate PTEN/Akt/HIF-1α pathway to inhibit glycolysis, and reverse 5-FU resistance under hypoxic conditions	Chen et al. (2015)
*In vitro* /Baicalin/0,4,7,11uM/−/−	AGS	Control,4uM,7uM,11uM,MS,MS + BA4uM MS + BA7uM,MS + BA11uM	NF-κB-NLRP3↓	Holds the prowess to promote gastric cancer cell pyroptosis by activating the NF-κB signaling pathway	Liu J. et al. (2024)
*In vitro*/Baicalin/80,120,160 μmol/L	MGC-803, BGC-823	Control, 80 μmol/L,120 μmol/L,160 μmol/L	Bax↑, Bcl-2↓, caspase-3↑, caspase-9↑	Induce GC cell apoptosis	Wang et al. (2017)
*In vitro*/Baicalin/6 μmol/L (AGS) and 12 μmol/L (SGC-7901)	AGS, SGC-7901	control, 5-Fu alone, Baicalin alone, Baicalin with Fer-1, and Baicalin with 5-Fu. Fer-1	ROS↓, TFR1↑, NOX1↑, COX2↑, FTH1↓, FTL↓, GPX4↓	Increase ROS in GC, promote ROS-dependent ferroptosis, enhance 5-Fu in GC, inhibit resistance	Yuan et al. (2023)
*In vitro* + *In vivo*/Berberine/0,10,25,50,75,100μM, 10 mg/kg/-/18d	BGC-823	0μM, 10μM, 25μM, 50μM, 75μM, 100μM, control, positive control group, and experimental group	Caspase3↑, mitochondrial membrane potential↑, p-AKT↓, p-mTOR↓, p70S6↓, S6 ↓	Regulate AKT-related mitochondrial pathway to induce GC cell apoptosis	Yi et al. (2015)
*In vitro*/Berberine/0, 25, 50, 100, and 200 μmol/L/−/−	SNU-5	0 μmol/L (control), 25 μmol/L, 50 μmol/L, 100 μmol/L, and 200 μmol/L	p53↑, Wee1 ↑, CDk1↑, Bax↑, Bcl-2 ↓	Induce p53 expression to decrease mitochondrial membrane potential, release cytochrome C, and activate caspase-3, induce apoptosis	Lin et al. (2006)
*In vitro* + *In vivo*/Berberine/0,5,10,20 mg/kg/normal saline with 0.5% DMSO/14d	BGC-823	control group,BBR5, 10 or 20 mg/kg	p-mTOR↓, p-p70S6K↓, p-Akt↓, p-ERK↓, p-JNK ↓, p-p38 ↓	Suppress mTOR, Akt, and MAPK (ERK, JNK, and p38) pathways to induce autophagy	Zhang et al. (2020)
*In vitro*/Berberine/10,20,40uM/−/−	MGC803, SGC7901,HepG2	Control, 10uM,20uM,40uM	N-cadherin↓, Vimentin↓, MMP-9↓, Snail↓, Slug↓, TGF-β, p-Smad2↓, β-catenin↓, p-Akt↓, p-PI3K↓	Berberine binds to TGF β R and regulates the TGFβ/Smad pathway to inhibit the EMT of GC cells	Du et al. (2021)
*In vitro* + *In vivo*/Berberine/20,30 μM,100 mg/kg/day/-/18d	AGS, SGC-7901	Control group; B20(Berberine 20 μM); B30(Berberine 30 μM); M20 (Metformin 20 μM); M3 (Metformin 30 μM); control group; BBR group; MET group	HNF4α↓, WNT5A↓, β-catenin↓	Regulate the AMPK/HNF4α/WNT5A signaling pathway to inhibit GC cell proliferation, invasion, and migration	Hu et al. (2018)
*In vitro* + *In vivo*/Berberine/0,10,20,40 μg/mL/50,100,150 mg/kg/-/27d	MKN-45, HGC-27	0 μg/mL, 10 μg/mL, 20 μg/mL, 40 μg/mL, Normal control, Tumor control, BBR-L (50 mg/kg), BBR-M (100 mg/kg), BBR-H (150 mg/kg) and S-1 (15 mg/kg)	p-JAK2↓, IL6↓, p-STAT3↓, BAX↑, BCL2↓, MMP9↓, P21↑, cyclinD1 ↓	Regulate the IL-6/JAK2/STAT3-related signaling pathway to inhibit GC cell proliferation	Xu et al. (2022)
*In vitro*/Berberine/40μM/−/−	AGS, MKN45	Control groups and 40 μM Berberine groups	ROS↑, MDA↑, SOD↓, Nrf2/HO-1↓, HIF-1α↓, EMT ↓	Regulation of oxidative stress may be one of the key mechanisms by which berberine inhibits the progression of GC	Han et al. (2025)
*In vitro* + *In vivo*/Berberine/30 μM,10 mg/kg/day/-/30d	SGC-7901, BGC-823	Blank, DDP (30 μM), Berberine (30 μM), DDP (30 μM)+Berberine (30 μM), Blank, DDP (3 mg/kg), berberine (10 mg/kg), DDP (3 mg/kg)+Berberine (10 mg/kg)	Cleaved caspase-3↑, cleaved caspase-9↑, Bax↑, MRP1↓, p-PI3K↓, p-AKT↓, mTOR-	Enhance DDP sensitivity possibly by reducing drug transporter proteins and inhibiting PI3K/AKT/mTOR signaling pathway	Kou et al. (2020)
*In vitro*/Berberine/10μM/−/−	SGC-7901/DDP, BGC-823/DDP	SGC-7901, SGC-7901+DDP, SGC-7901+BER, BGC-823, BGC-823+DDP, BGC-823+BER	miR-203↑, Bcl-w↓	Reduce cisplatin resistance by modulating the miR-203/Bcl-w apoptotic axis	You et al. (2016)
*In vitro* + *In vivo*/Berberine/20 μM,150 mg/kg/-/18d	MGC-803, GSK3β inhibitor	10 mM Glu, 10 mM Glu + BBR, 1.25 mM Glu, 1.25 mM Glu + BBR, Control, BBR, FAST, BBR/FED, BBR/FAST	MCL-1↓, BCL2↓, BAX↑, cleaved caspase3↓, p-pp2a↓, p-GSK3β↓	Berberine/low glucose combination inhibits GC growth via PP2A/GSK3β/MCL-1 signaling pathway	Peng et al. (2022)
*In vivo*/Berberine/100 mg/kg/18d/−/−/	MGC803, SGC7901	Control group, BBR group	HNF4α↓, WNT5α↓, β-catenin↓	Target HNF4α to delay GC subcutaneous xenograft growth	Li et al. (2022)
*In vitro* + *In vivo*/Berberine/50 mg/kg/-/4w	SGC-7901, BGC-823	Control, BBR, Erlotinib, Erlotinib + BBR	Bcl-xL↓, CyclinD1↓, EGFR-, p-EGFR↓, cleaved-PARP↑, p-AKT↓, AKT-, p-ERK↓, ERK-, pSTAT3↓, STAT3-, pNFκB↓, NFκB-, Bcl-xL↓	Berberine combining EGFR-TKIs enhances EGFR inhibition	Wang et al. (2016)
*In vitro*/Wogonoside/50 μM	AGS, MKN-45	Control, Wog (50 μM)	cleaved caspase-3↑, −9↑, Bax↑, Bcl-2↓, IRE1α↑, TRAF2↑, ASK1↑	Induce ER stress-related cell death via the IRE1α-TRAF2-ASK1 pathway, exhibiting anti-tumor activity	Gu et al. (2021)
*In vitro* + *In vivo*/Wogonin/60,90μM, 60 mg/kg/d/-/12d	SGC-7901, BGC-823	Untreated, DMSO, 60μM, 90μM, Control groups and Wogonin groups	JAK-STAT3	Exert antitumor effects in GC cells by downregulating the JAK-STAT3 pathway	Song et al. (2024)
*In vitro* + *In vivo*/Wogonin/50 µmol/L12.5 ng,/-/5d	BGC-823, Zebrafish	Control, Oxaliplatin, Wogonin, WOG + OXA	p-JNK↓, p-ULK1↓, LC3II↑, Mitochondrial Membrane Potential ↑	Induce nitrosative stress and autophagy, and enhance oxaliplatin chemotherapy	Hong et al. (2018)
*In vitro* + *In vivo*/Wogonin/60 mg/kg,20,30,50 μmol/L/−/−	BGC-823, MGC-803, MKN-45, HGC-27	control group; WOG60 mg/kg group; PTX10 mg/kg group; PTX20 mg/kg group; CDDP3mg/kg group; CDDP6mg/kg group; WOG (60 mg/kg)+PTX (10 mg/kg) group; WOG (60 mg/kg)+PTX (20 mg/kg) group; WOG (60 mg/kg)+CDDP (3 mg/kg) group; WOG (60 mg/kg)+ CDDP (6 mg/kg) group	-	Increase sensitivity to low-dose paclitaxel chemotherapy by inhibiting GC cell proliferation	Wang et al. (2013)
*In vitro* + *In vivo*/Wogonin/100 μM,15,30,60 mg/kg/day/-/2w	MGC-803	Control,5-FU, WOG, 5-FU + WOG; Negative Control group); WOG 60, 30, 15 mg/kg/day group; 5-FU 20, 10 mg/kg/day group; WOG + 5-FU (10 mg/kg) combination group	-	Sensitize GC cells to 5-FU-induced apoptosis by inhibiting NF-κB nuclear translocation	Zhao et al. (2010)
*In vitro*/Wogonin/15 μg/mL/−/−	SGC-7901	Control group, Wogonin group	MCT-4↓, HIF-1α↓, LDH↓, SDH↓, ATP↓	Inhibit GC cell proliferation, energy metabolism, and angiogenesis	Wang S. J. et al. (2019)
*In vitro* + *In vivo*/Wogonin/0,50,100,200 µM/-/2w	MFC	Ctrl siRNA, PERK siRNA,0 h, 1 h,2 h,6 h,12 h,24 h, Control group, Wogonin group, Control (24 h),50 µM(24 h),100 µM(24 h),200 µM(24 h)	PERK↑, AKT↓, p-AKT↓, HMGB1↑, P38↑, JNK	Induction of calreticulin (CRT) and membrane-associated protein 1 translocation to the cell membrane, as well as release of high mobility group box 1 (HMGB1) and ATP to trigger effective anti-tumor immune response	Yang et al. (2012)
*In vitro* + *In vivo* /Glycyrrhizin/TRAIL (80 ng/mL) LIQ (50 uM)/−/−	AGS, SNU-216, nude mice	control group, treatment group (TRAIL 100 μg/mouse)	TRAIL, E-cadherin↑, N-cadherin↓, Vimentin↓, Twist↓, cleaved caspase8,9↑, cleaved PARP↑, p-PARP-, BCL-2↓, BAX↑, FADD↑, DR4↑, DR5↑, JNK-, p-JNK↑, ROS↑	Synergistically promote cell apoptosis and ROS generation, inhibit growth of human GC cells and subcutaneous transplant tumors in nude mice	Xie et al. (2017)
*In vitro* + *In vivo*/Glycyrrhizin/80 μM,15 mg/kg/-/21d	SGC-7901/DDP, *in vivo*	Control, LIQ, DDP, LIQ + DDP	Cleaved caspase-8/-9/-3↑, cleaved PARP↑, LC3B↑, Beclin 1↑	Synergistically enhance cisplatin chemotherapy with glycyrrhizin	Wei et al. (2017)
*In vitro* + *In vivo*/Glycyrrhizin/5, 10, and 20 μM/-/18d	MGC-803	control group,10 mg/kg of LCD, 20 mg/kg of LCD, 20 mg/kg of 5-FU.	ICMT↓, Bcl-2↓, bax↑, cytc↓, caspase3↑, cyclinD1↓, CDK4↓	Promote GC cell apoptosis via the ICMT/Ras pathway, inhibit migration and invasion	Ma et al. (2022)
*In vitro*/Glycyrrhizic acid/1 mg/mL/−/−	MGC-803, BGC-823, SGC-7901	Control, GA (1 mg/mL)	Bcl-2↓, survivin↓, p65↓, PI3K↓, p-PI3K↓, AKT-, p-AKT↓	Induce cell cycle arrest and apoptosis, inhibit the PI3K/AKT signaling pathway	Wang et al. (2020)

### 5.2 Inducing GC cell autophagy

Autophagy and apoptosis are interconnected cellular processes that share multiple regulatory factors and signaling components (Zhang et al., 2023). Functional crosstalk exists between these two pathways, whereby modulation of one process can significantly influence the other (Woo et al., 2020). For instance, inhibition of apoptosis may suppress autophagy, while activation of apoptotic signaling can also trigger autophagic responses (Hu et al., 2023).

The PI3K/AKT/mTOR pathway is a central regulatory node for both autophagy and cell survival. Inhibition of this pathway typically promotes autophagy and sensitizes cells to apoptosis. The tumor suppressor PTEN acts as a key positive regulator of autophagy by antagonizing PI3K/AKT signaling (Yamada and Araki, 2001). Consequently, PTEN status (e.g., deletion or mutation) can influence cellular sensitivity to therapeutic agents (Álvarez-Garcia et al., 2019). Other important pathways involved in this crosstalk include the AMPK and MAPK signaling cascades, both of which converge on mTOR and other downstream targets to modulate autophage and apoptosis.

Several bioactive metabolites in BXD modulate both autophage and apoptosis in GC. BXD exerts anti-tumor effects through the AMPK/PTEN/HSP90 axis, with notable efficacy in AGS cells (Jian et al., 2016). Berberine induces autophagy in GC cells through the MAPK/mTOR/p70S6K and AKT signaling pathways (Zhang et al., 2020). Baicalein induces a late-stage blockade of autophagic flux and inhibits GC proliferation by upregulating miR-7, which in turn inhibits the PI3K/AKT/mTOR pathway (Wen et al., 2024) (See Table 2).

### 5.3 Inhibition of GC cell migration

Bioactive metabolites exert potent anti-metastatic effectis in GC metastasis by targeting multiple critical biological processes. A primary mechanism is the inhibition of EMT, a key process in cancer metastasis marked by downregulation of E-cadherin and upregulation of N-cadherin and vimentin (Jiang, 2018; Li et al., 2018). Baicalein inhibits EMT through suppression of the TGF-β/Smad4 (Chen et al., 2014) and p38 (Yan et al., 2015) signaling pathways, as well as by downregulating p12-LOX-mediated EMT and the MEK/ERK pathways (Ye et al., 2024). Berberine reverses EMT by targeting the TGF-β pathway (Du et al., 2021) and modulates oxidative stress through suppression of the Nrf2/HO-1 and HIF-1α pathways (Han et al., 2025).

Furthermore, these metabolites suppress cell invasion and migration by disrupting the extracellular matrix (ECM) remodelling and related signaling. Berberine demonstrates potent anti-migratory activity by regulating both the AMPK/HNF4α/WNT5A (Hu et al., 2018) and IL-6/JAK2/STAT3 (Xu et al., 2022) signaling pathways. Wogonin suppresses metastasis by inhibiting the JAK-STAT3 signaling pathway (Song et al., 2024).

In addition, certain metabolites inhibit angiogenesis, the formation of new blood vessels essential for tumor growth and metastasis. For instance, baicalein suppresses angiogenesis by activating miR-7, which in turn inhibits the FAK/AKT pathway (Qiao et al., 2022) (see Table 2).

### 5.4 Reducing drug resistance in GC cells

Reducing chemoresistance remains a major challenge in GC treatment. Several bioactive metabolites from BXD have demonstrated potential to enhance chemosensitivity through diverse mechanisms.

Berberine modulates multiple pathways to overcome drug resistance. It induces apoptosis by regulating apoptosis-related proteins (Wang et al., 2017) and promotes ROS-dependent ferroptosis to enhance sensitivity to 5-Fu (Yuan et al., 2023). Furthermore, berberine increases cisplatin sensitivity by downregulating drug efflux transporters (MDR1, MRP1), inhibiting the PI3K/AKT/mTOR pathway (Kou et al., 2020) and modulating the miR-203/Bcl-w axis (You et al., 2016).

Flavonoids from *Scutellaria baicalensis* also exhibit chemosensitizing effects. Baicalin enhances cisplatin sensitivity in SGC-7901/DDP cells by regulating the Nrf2/Keap1 pathway (Li et al., 2020) and reverses 5-FU resistance under hypoxic conditions by suppressing glycolysis via the PTEN/Akt/HIF-1α pathway (Chen et al., 2015).

Wogonoside enhances the efficacy of oxaliplatin by inducing nitrosative stress and autophagy (Hong et al., 2018), increases sensitivity to low-dose paclitaxel (Wang et al., 2013), and promotes 5-FU-induced apoptosis by inhibiting NF-κB nuclear translocation (Zhao et al., 2010). Glycyrrhizin enhances cisplatin sensitivity by modulating autophagy to promote apoptosis (Wei et al., 2017) (see Table 2).

### 5.5 Improving metabolism

Glycogen synthase kinase 3β (GSK3β), a conserved serine/threonine kinase, is a key regulator in GC cell proliferation and metabolic reprogramming (Pan et al., 2019). It integrates signaling from pathways such as Wnt and AKT. Normally, GSK3β phosphorylates β-catenin, marking it for degradation. However, AKT-mediated phosphorylation inactivates GSK3β, leading to β-catenin stabilization, nuclear translocation, and subsequent activation of pro-proliferative genes (e.g., c-myc, Cyclin D1) (Wu et al., 2016; Zhu and Li, 2023). Wogonin inhibits GC proliferation, angiogenesis, and energy metabolism by targeting the GSK3β/β-catenin axis (Wang S. J. et al., 2019).

Several bioactive metabolites in BXD disrupt tumor metabolism in GC. Berberine blocks cellular energy supply and, under low glucose conditions, inhibits GC progression via the PP2A/GSK3β/MCL-1 pathway (Peng et al., 2022). It also modulates the AMPK–HNF4α–WNT signaling axis, affecting metabolic reprogramming and apoptosis via regulation of HNF4α and EGFR (Li et al., 2022; Wang et al., 2016) (see Table 2).

### 5.6 Immunomodulation

Wogonin exerts immunomodulatory anti-cancer effects through two distinct mechanisms (Yang et al., 2012). First, it alleviates hypoxia-induced immunosuppression by downregulating TGF-β1 in GC cells, which subsequently suppresses the induction of regulatory T cells (Tregs) (Yao et al., 2025). Second, under the conditions of endoplasmic reticulum stress, wogonin modulates the PI3K pathway to induce ICD, a process mediated by the release of key immunostimulatory signals (Huynh et al., 2020). These findings establish wogonin as a promising immunotherapeutic agent capable of both inhibiting tumor immune evasion and actively stimulating anti-cancer immune response (see Table 2).

## 6 Discussion

This review highlights the promising potential of BXD and its bioactive metabolites as multi-target therapeutic agents for GC. Unlike standard chemotherapeutics, which are hampered by high toxicity, drug resistance, and limited patient applicability, BXD exerts anti-GC effects through coordinated modulation of diverse molecular pathways. GC exhibits high heterogeneity as defined by Lauren classification (intestinal type vs. diffuse type) and TCGA molecular subtypes (EBV-positive, MSI, CIN, and GS), accompanied by pronounced stromal reaction, hypoxia, acidic microenvironment, and a strong propensity for peritoneal metastasis (Li et al., 2026). The diffuse type, particularly signet-ring cell carcinoma, is more prone to peritoneal dissemination. Tumor cells can reshape peritoneal mesothelial cells and the stroma through exosomes to create a pre-metastatic niche (Abe and Ushiku, 2022). Thus, the multi-target and multi-pathway characteristics of BXD confer distinct advantages in GC treatment.

BXD inhibits the proliferation of GC cells by inducing apoptosis, autophagy, and metabolic suppression. BXD and its active metabolites simultaneously and potently inhibit multiple parallel pro-tumorigenic signaling hubs, particularly the PI3K/AKT/mTOR and Wnt/β-catenin pathways, thereby effectively blocking the ability of tumor cells to evade growth suppression. On this basis, BXD not only initiates the classical apoptotic program by increasing the Bax/Bcl-2 ratio, activating the caspase cascade, and inducing ERS, but also modulates autophagy through the AMPK/PTEN/HSP90 axis and even triggers pyroptosis via the NF-κB-NLRP3 pathway. Notably, the active metabolites of BXD such as berberine and baicalin interfere with the metabolic reprogramming of GC cells and disrupt the energy supply via glycolysis suppression by regulating the PP2A/GSK3β axis and HIF-1α under hypoxic or hypoglycemic conditions.

BXD inhibits EMT to reduce the invasive and metastatic potential of GC cells, with particular potential significance for suppressing peritoneal metastasis (PM). Invasion and metastasis are the leading causes of death in GC patients, and PM remains a major clinical challenge due to inadequate targeted therapies. BXD demonstrates unique potential in frontier intervention against metastasis. The heterogeneity and high metastatic rate of GC are primary reasons for its poor prognosis, and EMT serves as a critical step in metastasis, characterized by downregulation of E-cadherin and upregulation of N-cadherin and vimentin (Jiang, 2018; Li et al., 2018). BXD downregulates lncRNA TUC338 to inhibit EMT, invasion, and migration (Dai et al., 2023). Baicalin reverses EMT through the TGF-β/Smad4, p38, and 12-LOX/MEK/ERK pathways (Chen et al., 2014; Yan et al., 2015; Ye et al., 2024), berberine targets TGF-β, Nrf2/HO-1, and HIF-1α to regulate oxidative stress and disrupt the extracellular matrix (ECM) (Du et al., 2021; Han et al., 2025), and glycyrrhizin inhibits migration via the AMPK/HNF4α/WNT5A and IL-6/JAK2/STAT3 pathways (Hu et al., 2018; Xu et al., 2022). Particularly, BXD interferes with exosome-induced EMT in peritoneal mesothelial cells derived from GC cells, thereby disrupting the formation of the pre-metastatic microenvironment (Li et al., 2023; Ng et al., 2025; Sun et al., 2022), which is especially critical in advanced GC as it may reduce the incidence of distant metastasis.

Chemoresistance represents a major obstacle to the success of adjuvant therapy in GC. BXD shows significant advantages in sensitizing chemotherapeutic agents such as cisplatin, 5-FU, and oxaliplatin and reversing drug resistance. Its mechanisms extend beyond the traditional downregulation of drug efflux pumps (e.g., MDR1 and MRP1) to include regulation of ROS-dependent ferroptosis, induction of nitrosative stress, and modulation of the miR-203/Bcl-w non-coding RNA axis. Furthermore, the immunomodulatory effects of BXD suppress tumor immune escape, enhance immune surveillance, and improve the efficacy and resistance-reversal potential of chemotherapy or targeted therapies. The GC tumor microenvironment is enriched with immunosuppressive cells, such as PMN-MDSCs, which promote tumor progression and metastasis by secreting soluble factors (Gabrilovich and Nagaraj, 2009; Hu et al., 2022). BXD downregulates PD-L1 expression and modulates MGMT activity through the IL-6/JAK/STAT3 pathway to enhance cisplatin sensitivity (Feng et al., 2021b) and it inhibits PD-L1-induced apoptosis through multiple targets (Feng et al., 2021a). Baicalein alleviates hypoxia-induced Treg generation by downregulating TGF-β1 and activates the PI3K pathway to trigger ICD and release damage-associated molecular patterns (DAMPs) (Yang et al., 2012). In addition, berberine promotes ROS-dependent ferroptosis to reverse 5-FU resistance (Yuan et al., 2023), baicalin enhances cisplatin sensitivity by regulating the Nrf2/Keap1 pathway (Li et al., 2020), and oroxylin A induces nitrosative stress and autophagy to synergize with oxaliplatin (Hong et al., 2018). These effects strengthen immune surveillance (e.g., enhancing NK cell and CD8^+^ T cell activity) and reverse multidrug resistance through the autophagy-apoptosis axis and miR-203/Bcl-w regulation (You et al., 2016), along with downregulation of MDR1/MRP1 (Kou et al., 2020). This provides a sensitization window for combination with PD-1 inhibitors or EGFR-targeted agents, potentially improving the objective response rate (ORR) and progression-free survival (PFS) in patients with advanced GC.

Considering the inflammatory microenvironment and high PD-L1 expression associated with immune escape in GC (Zhang et al., 2024), the immunomodulatory effects of BXD can serve as a pivot for combination therapy with chemotherapy, targeted therapy, and immunotherapy as BXD acts as a natural modulator targeting these pathological features. The active metabolites in BXD potently intervene in the IL-6/JAK/STAT3 pathway, the central hub linking inflammation and cancer, thereby blocking the cascade amplification of pro-inflammatory factors and directly leading to downregulation of PD-L1 expression. This finding holds significant clinical promise because the monotherapy response rate of PD-1/PD-L1 immune checkpoint inhibitors (ICIs) in GC remains limited (De Mello et al., 2019). Therefore, utilizing the TME-remodeling effects of BXD as a pivot in combination with existing chemotherapy, targeted therapy, and immunotherapy offers a novel integrative Chinese-Western medicine approach to overcome immune tolerance and achieve long-term survival benefits in GC patients. Although studies on BXD and its major active metabolites such as baicalin, baicalein, berberine, and wogonin have confirmed its anti-GC effects, the safety profile of BXD in GC treatment has not been fully elucidated. This review only explored the mechanisms of action of some identified active metabolites of BXD. It is noteworthy that the herbal decoction may contain thousands of metabolites that act on multiple molecular pathways. Consequently, certain active metabolites from individual botanical drugs within the formula may not have been fully detected, representing a major challenge in metabolite formula research. Therefore, further identification of the effective metabolites of BXD and their anti-GC activities using more advanced analytical techniques remains a key focus for future research.

## 7 Limitations and further perspective

The therapeutic efficacy of BXD arises from the synergistic actions of its multiple herbal metabolites and thousands of potential phytochemical metabolites. Current research has primarily focused on a few well-characterized metabolites such as baicalin, berberine, and oroxylin A. However, other trace constituents, potential novel metabolites, and the phytochemical interactions within the formula (e.g., the formation of insoluble complexes during decoction) have not been sufficiently investigated. Some flavonoid and alkaloid metabolites in BXD (e.g., berberine and baicalein) may exhibit PAINS (pan-assay interference metabolites) characteristics in certain screening models (Jasial et al., 2017), necessitating cautious interpretation of conclusions drawn solely from *in vitro* high-throughput screening. At present, *in vivo* pharmacokinetic studies of BXD still remain limited. The absorption, distribution, metabolism, and excretion (ADME) profiles of its active metabolites, particularly the secondary metabolites generated through intestinal microbiota transformation after oral administration, require substantial further investigation. The lack of reliable PK/PD data hinders the scientific optimization and individualization of clinical dosing regimens of BXD. The multi-component and multi-target nature of BXD is both an advantage and a significant analytical challenge. Advanced phytochemical analysis techniques, such as ultra-performance liquid chromatography-quadrupole time-of-flight tandem mass spectrometry (UPLC-QTOF-MS/MS), metabolomics, transcriptomics, proteomics, and single-cell sequencing should be employed in future studies to elucidate the synergistic interactions among different metabolites and to uncover the integrative medicinal mechanisms by which BXD exerts its therapeutic effects as a whole. Although the potential of BXD in immune regulation, angiogenesis inhibition, and peritoneal metastasis suppression has been highlighted, most existing studies remain focused on its direct effects on tumor cells. The TME of GC is highly complex, involving immune cells, fibroblasts, extracellular matrix, and multiple other components, all of which are closely associated with tumor occurrence and development (Yan et al., 2017). However, current mechanistic studies on BXD intervention in GC rarely involve key TEM components, including macrophages, exosomes, and mesenchymal stem cells (Lee, 2026) Macrophages can assist tumor cells in evading immune surveillance to promote the systemic spread of tumor cells. Mesenchymal stem cells contribute to tumor progression by secreting growth factors and extracellular matrix components, creating a supportive niche that facilitate tumor cell proliferation, metastasis and infiltration (Panzetta et al., 2025). Additionally, tumor cells can remodel the TME through exosomes, and the stressful TME in turn regulates the release of exosomes, affecting the remodelling and eading to tumor progression (Eso et al., 2020). How BXD systematically reshapes this highly complex TME—for instance, how it dynamically modulates the infiltration and functional states of different immune cell subpopulations such as M1/M2 macrophages, MDSCs, and regulatory Tregs, and how it regulates cancer-associated fibroblasts to inhibit stromal remodeling—remains to be fully elucidated. Future studies should employ more advanced experimental models to evaluate the differential therapeutic efficacy of BXD across various molecular subtypes of GC. In particular, the establishment of orthotopic peritoneal metastasis models will be valuable for investigating how BXD intervenes in the tumor exosome–peritoneal mesothelial cell axis to block the formation of the pre-metastatic niche.

Current clinical studies on BXD are predominantly small-sample, non-randomized controlled trials with relatively low levels of evidence, mainly focus on auxiliary endpoints such as improvement of quality of life and alleviation of chemotherapy-related adverse effects. Robust evidence supporting BXD’s ability to prolong key endpoints such as overall survival and PFS remains insufficient. More importantly, existing research has rarely performed stratified analyses based on the high heterogeneity of GC, such as Lauren classification or TCGA molecular subtypes. Large-scale, multicenter, randomized, double-blind, placebo-controlled clinical trials are urgently needed to generate high-level evidence-based medical data, and trial designs should incorporate molecular subtyping to enable precise patient stratification. The anti-resistance mechanisms of BXD offer new opportunities for the clinical management of GC. Future research should employ biomarker-guided precision strategies to conduct both preclinical and clinical trials investigating the combination of BXD with conventional chemotherapy, targeted agents, and especially immune checkpoint inhibitors. The ultimate goal is to achieve the clinical objective of potentiating efficacy while attenuating toxicity.

## 8 Conclusion

BXD is a valuable therapeutic agent for GC, exhibiting significant preventive and therapeutic effects through its multifaceted mechanisms of action. Clinically, BXD serves as an effective adjuvant to chemotherapy, ameliorates postoperative complications, and improves patient quality of life. Mechanistically, BXD exerts anti-GC effects by inducing apoptosis, suppressing EMT to inhibit cell migration and invasion, remodeling the TME, and reversing chemoresistance. These diverse effects are mediated by its active metabolites, offering potential for targeted interventions. Therefore, BXD represents a promising complementary and integrative therapeutic strategy for GC. However, further rigorous investigation and clinical validation are warrented to fully elucidate its mechanisms.
